# The Impact of Type 2 Myocardial Infarction in Acute Pancreatitis: Analysis of 1.1 Million Hospitalizations and Review of the Literature

**DOI:** 10.7759/cureus.44113

**Published:** 2023-08-25

**Authors:** Tejasvini Khanna, Jay Patel, Ishandeep Singh, Shivam Kalra, Mukul Dhiman, Isha Kohli, Hunza Chaudhry, Dino Dukovic, Aalam Sohal, Juliana Yang

**Affiliations:** 1 Medical School, Maulana Azad Medical College, New Delhi, IND; 2 Internal Medicine, Orange Park Medical Center, Orange Park, USA; 3 Internal Medicine, Dayanand Medical College and Hospital, Punjab, IND; 4 Internal Medicine, Dayanand Medical College and Hospital, Ludhiana, IND; 5 Internal Medicine, Punjab Institute of Medical Sciences, Punjab , IND; 6 Public Health Sciences, Icahn School of Medicine at Mount Sinai, New York, USA; 7 Internal Medicine, University of California, San Francisco, USA; 8 Internal Medicine, Ross University School of Medicine, Bridgetown, BRB; 9 Hepatology, Liver Institute Northwest, Seattle, USA; 10 Gastroenterology and Hepatology, University of Texas Medical Branch, Galveston, USA

**Keywords:** acute kidney injury, deep vein thrombosis, pulmonary embolism, sepsis, mortality, myocardial infarction type 2, acute pancreatitis

## Abstract

Introduction

Acute pancreatitis (AP) is a common inflammatory disorder with acute onset and rapid progression. Studies have reported cardiac injury in patients with AP. It is often thought that stress cardiomyopathy can induce these changes leading to type 2 myocardial infarction (type 2 MI) in AP. Our study aims to assess the prevalence as well as the impact of type 2 MI on outcomes in patients with AP.

Methods

National Inpatient Sample (NIS) 2016-2020 was used to identify adult patients (age>18) with acute pancreatitis. We excluded patients with STEMI, NSTEMI, pancreatic cancer, or chronic pancreatitis. Patients with missing demographics and mortality were also excluded. Patients were stratified into two groups, based on the presence of type 2 MI. Multivariate logistic regression analysis was performed to assess the impact of concomitant type 2 MI on mortality, sepsis, acute kidney injury (AKI), ICU admission, deep venous thrombosis (DVT), and pulmonary embolism (PE) after adjusting for patient demographics, hospital characteristics, etiology of AP and the Elixhauser comorbidities.

Results

Of the 1.1 million patients in the study population, only 2315 patients had type 2 MI. The majority of the patients in the type 2 MI group were aged >65 years (49.2%, p<0.001), males (54.6%, p=0.63), White (67.6%, p=0.19), had Medicare insurance (55.5%, p<0.001), and were in the lowest income quartile (34.8%, p=0.12). Patients in the type 2 MI group had a higher incidence of mortality (5.4% vs 0.6%, p<0.001), sepsis (7.1% vs 3.7%, p<0.001), shock (9.3% vs 0.9%, p<0.001), AKI (42.9% vs. 11.8%, p<0.001) and ICU admission (12.1% vs 1.4%, p<0.001). After adjusting for confounding factors, patients in the type 2 MI group were noted to be at higher odds of mortality (aOR=2.4; 95% CI 1.5-3.8, p<0.001). Patients in the type 2 MI group had a longer length of stay (adjusted coefficient=2.1 days; 95% CI 1.4-2.8; p<0.001) and higher total hospitalization charges (adjusted coefficient=$45,088; 95% CI $30,224-$59,952; p<0.001).

Conclusion

Although the prevalence of type 2 MI in AP is low, the presence of type 2 MI is associated with increased mortality and worse outcomes. Physicians should be aware of this association and these patients should be monitored carefully to prevent worse outcomes.

## Introduction

The universal definition of myocardial infarction (UDMI) has delineated five subtypes of myocardial infarction (MI) [1]. While type 1 MI is generally attributable to an atherothrombotic plaque, type 2 MI refers to ischaemic injury secondary to a discrepancy in oxygen supply and demand [1]. Type 2 MI has mostly been described in hospitalized, older patients with multiple comorbidities [2]. It has many causes, ranging from anemia and sepsis to cardiac events such as tachyarrhythmia. Possibly, tachycardia triggers type 2 MI by causing an increase in myocardial work as well as reducing coronary perfusion by decreasing the duration of diastole, thereby creating a supply-demand mismatch [3]. Patients with type 2 MI may have minimal to no symptoms or ECG changes, and generally present with lower troponin levels than type 1 MI [3].

Acute pancreatitis (AP), a disease involving inflammation and necrosis of the pancreas, is a major cause of emergency visits and hospital admissions from gastrointestinal disease [4]. Given the significant morbidity and mortality that AP carries, it is necessary to understand the factors that significantly determine its prognosis. Various studies have identified abnormal cardiovascular findings in patients of AP [5,6]. ECG changes, including tachyarrhythmias, bradyarrhythmias, atrial flutter, atrial fibrillation, supraventricular premature contractions, short PR interval, QRS prolongation, bundle-branch blocks, T-wave flattening, and ST-segment depressions, have been observed in 25-50% patients of AP [7,8]. Another study found that over 35% of AP patients had elevated levels of hs-TnT [9]. Previously, a cohort study also reported a reverse association, in that diabetic patients with a history of AP had a higher incidence of stroke and MI in the future [10]. Although the mechanism of these changes is unclear, proposed explanations include hypovolemia, electrolyte disturbances, hemodynamic instability, proteolytic enzymes released by the pancreas, and myocardial ultrastructural disturbances [11]. The release of inflammatory mediators in AP may also be responsible for increasing myocardial permeability or inducing coronary vasospasm. Electrocardiogram abnormalities such as T-wave flattening and ST-segment depression have been described [11,12]. More recently, increases in troponin and brain natriuretic peptide (BNP) levels have been reported [7,13].

There is an ongoing effort to study whether evidence of cardiovascular injury indicates increased severity of AP. Concomitant AP and MI are theorized to carry a poor prognosis [14]. Researchers have found an association between high-sensitivity troponin-T (hs-TnT) levels and the severity of AP [7]. Contrarily, a study tested the relationship between cardiac troponin-I (cTnI) and AP prognosis and reported no association [6]. Yet another group of researchers found that creatine kinase myocardial band (CK-MB) levels predicted severe AP, whereas cTnI or N-terminal pro-brain natriuretic peptide (NT-proBNP) did not [15]. Overall, it is unclear if abnormal cardiovascular findings portend a poor prognosis for AP. With this background, the following study was carried out to examine the association between type 2 MI and outcomes in AP.

## Materials and methods

Data source 

The National Inpatient Sample (NIS), maintained by the Healthcare Cost and Utilization Project (HCUP), is the largest database of inpatient hospital stays in the United States [16]. It contains information on 35 million weighted hospitalizations annually. Information regarding this data source has been discussed in previous studies [17,18]. Each hospitalization is de-identified and maintained in the NIS as a unique entry with one primary discharge diagnosis and up to 39 secondary diagnoses during that hospitalization, depending on the year of data collection. Each entry carries patient demographics, including age, sex, race, insurance status, primary and secondary procedures (up to 25), hospitalization outcome, total charges, and length of stay (LOS). IRB approval was not required as this study was conducted on publicly available de-identified data.

Study population

**Figure 1:**
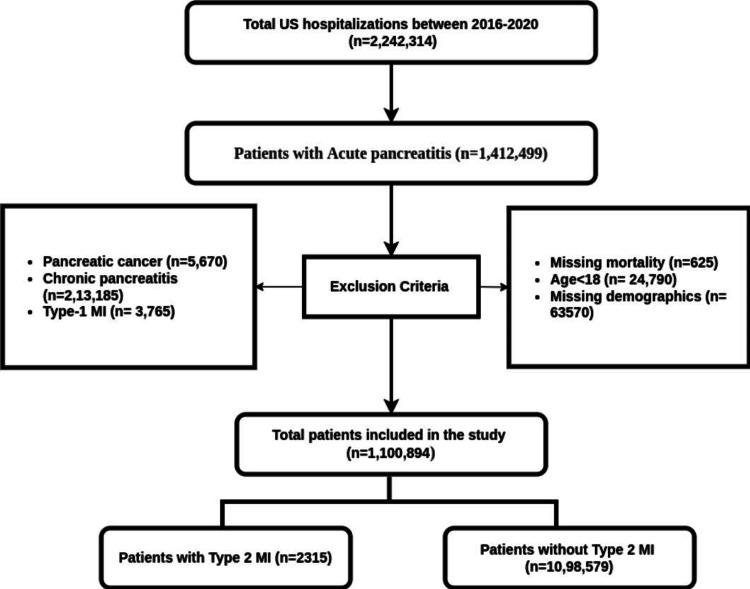
Selection of study population US: United States; MI: myocardial infarction

We used the International Classification of Diseases 10th Version, Clinical Modification (ICD-10 CM) diagnosis codes to identify adult patients hospitalized with a primary diagnosis of acute pancreatitis (AP) between 2016 and 2019. We excluded cases with missing data on in-hospital mortality, demographic information, or concomitant diagnosis of pancreatic cancer/chronic pancreatitis or acute type 1 myocardial infarction (AMI). In total, 1,100,894 cases met the inclusion criteria. Patients were stratified into two groups, based on the presence or absence of type 2 MI. This information is presented in Figure 1. 

Study outcomes and variables

The primary study outcome was inpatient mortality between patients with type 2 MI and those without. Secondary outcomes included sepsis, shock, AKI, and ICU admission. We also compared the mean LOS and total hospitalization charges between the two groups. Information was also collected on age groups (divided into three groups; <44 years, 45-64 years, and >65 years), gender, race, primary insurance, median income, and hospital characteristics (region, bed size, and rural/urban location). Data were also collected on the etiology of AP based on the presence or absence of diabetes. The Elixhauser comorbidity index was used to assess the burden of comorbidities [19]. This is a well-validated index based on ICD 10-CM codes meant to be used in extensive administrative data and has been shown to predict mortality in patients with AP [20]. 

Statistical analysis

National estimates were generated using hospital discharge weights provided by NIS. Chi-square and independent t-tests were used to compare categorical and continuous variables respectively. Univariate logistic regression was performed to identify the association between demand ischemia and categorical/continuous outcomes. Multivariate logistic regression was conducted while adjusting for patient demographics, hospital characteristics, and Elixhauser comorbidities for the variables that met the cut-off of p<0.1 on univariate analysis. The unadjusted and adjusted odds ratios were reported with a 95% confidence interval. A p-value <0.05 was considered statistically significant. STATA 17.0 (StataCorp LLC, College Station, TX) was used for data analysis.

## Results

Demographics 

Out of 1,098,580 hospitalizations for acute pancreatitis, 2,315 patients experienced Type 2 MI. Among those with Type 2 MI, most patients were over 65 years old (49.2%), were males (54.6%), identified as White (67.6%), and were primarily on Medicare (55.5%). The majority of the patients with Type 2 MI were in the lowest income quartile (34.8%). The data is further presented in Table 1.

**Table 1:** Patient demographics, hospital characteristics, and etiology of pancreatitis, stratified by the presence of type 2 MI MI: myocardial infarction

Demographics	Absence of Type 2 MI n (%)	Presence of Type 2 MI n (%)	p- value
Age Category (in years)			<0.001
18-44	393,420 (35.8)	275 (11.9)	
45-64	444,420 (40.4)	900 (38.9)	
>65	260,740 (23.7)	1,140 (49.2)	
Sex			0.63
Males	588,215 (53.5)	1,265 (54.6)	
Females	510,365 (46.4)	1,050 (45.4)	
Race			0.19
White	699,855 (63.7)	1,565 (67.6)	
Black	183,350 (16.7)	390 (16.8)	
Hispanic	149,115 (13.6)	225 (9.7)	
Asian/Pacific Islander	23,815 (2.2)	60 (2.6)	
Native American	9,465 (0.9)	10 (0.4)	
Other	32,980 (3)	65 (2.8)	
Primary Expected Payer			<0.001
Medicare	324,410 (29.5)	1,285 (55.5)	
Medicaid	264,065 (24)	365 (15.8)	
Private	359,910 (32.8)	460 (19.9)	
Uninsured	108,675 (9.9)	135 (5.8)	
Median Household Income			0.12
Lowest quartile	354,970 (32.3)	805 (34.8)	
Second quartile	293,540 (26.7)	600 (25.9)	
Third quartile	257,845 (23.5)	595 (25.7)	
Highest quartile	192,225 (17.5)	315 (13.6)	
Region of Hospital			0.1
Northeast	188,940 (17.2)	505 (21.8)	
Midwest	238,585 (21.7)	495 (21.4)	
South	451,335 (41.1)	880 (38)	
West	219,720 (20)	435 (18.8)	
Hospital Location			0.24
Rural	129,255 (11.8)	230 (9.9)	
Urban	969,325 (88.2)	2,085 (90)	
Teaching Status of Hospitals			0.003
Non-teaching hospitals	406,545 (37)	690 (29.8)	
Teaching hospitals	692,035 (63)	1,625 (70.2)	
Bed Size of Hospitals			<0.001
Small	281,495 (25.6)	490 (21.2)	
Medium	335,300 (30.5)	525 (22.7)	
Large	481,785 (43.9)	1,300 (56.2)	
Etiology			0.009
Biliary	217,280 (19.8)	570 (24.6)	
Alcohol	332,135 (30.2)	580 (25)	
Other	549,165 (50)	1,165 (50.3)	

Comorbidities 

Higher incidence of several comorbidities was found among type 2 MI patients as compared to those without type 2 MI: congestive heart failure (33.9% vs 6.5%, p<0.001), cardiac arrhythmias (34.3% vs 11.6%, p<0.001), valvular disease (10.6% vs 2.1%, p<0.001), pulmonary circulation disorders (7.9% vs 1.2%. p<0.001), peripheral vascular disorders (9.5% vs 3.5%, p<0.001), other neurological disorders (16.6% vs 5.5%, p<0.001), chronic pulmonary disease (21.6% vs 15.6%, p<0.001), complicated diabetes (24.4% vs 13%, p<0.001), hypothyroidism (14.9% vs 9.2%, p<0.001), renal failure (26.1% vs 8.8%, p<0.001), solid tumor without metastasis (3.4% vs 1.7%, p=0.006), coagulopathy (16.6% vs 7.3%, p<0.001), weight loss (12.5% vs 6.3%, p<0.001), fluid and electrolyte disorders (65.2% vs 41%, p<0.001), and deficiency anemia (6.3% vs 3.9%, p=0.008), and hypertension, complicated (40.8% vs 10.8%, p<0.001). Conversely, there was a lower incidence of uncomplicated hypertension (37.1% vs 45.8%, p<0.001) and alcohol abuse (27.9% vs 33.1%, p=0.018) in type 2 MI patients. These results are described in Table 2.

**Table 2:** Patient comorbidities, stratified by the presence of type 2 MI MI: myocardial infarction; AIDS/HIV: acquired immunodeficiency syndrome/ human immunodeficiency virus

Underlying comorbidity	Absence of Type 2 MI n (%)	Presence of Type 2 MI n (%)	p-value
Congestive heart failure	72,055 (6.5)	785 (33.9)	<0.001
Cardiac arrhythmias	127,720 (11.6)	795 (34.3)	<0.001
Valvular disease	23,100 (2.1)	245 (10.6)	<0.001
Pulmonary circulation disorders	13,265 (1.2)	185 (7.9)	<0.001
Peripheral vascular disorders	38,425 (3.5)	220 (9.5)	<0.001
Hypertension, uncomplicated	503,470 (45.8)	860 (37.1)	<0.001
Paralysis	2,520 (0.2)	5 (0.2)	0.95
Other neurological disorders	60,985 (5.5)	385 (16.6)	<0.001
Chronic pulmonary disease	171,545 (15.6)	500 (21.6)	<0.001
Diabetes, uncomplicated	153,205 (13.9)	340 (14.7)	0.64
Diabetes, complicated	142,820 (13)	565 (24.4)	<0.001
Hypothyroidism	101,140 (9.2)	345 (14.9)	<0.001
Renal failure	96,940 (8.8)	605 (26.1)	<0.001
Liver disease	234,700 (21.3)	535 (23.1)	0.36
Peptic ulcer disease excluding bleeding	17,255 (1.6)	60 (2.6)	0.07
AIDS/HIV	3,550 (0.3)	10 (0.4)	0.68
Lymphoma	3,830 (0.3)	15 (0.6)	0.27
Metastatic cancer	10,475 (0.9)	30 (1.3)	0.45
Solid tumor without metastasis	18,775 (1.7)	80 (3.4)	0.006
Rheumatoid arthritis/collagen vascular disease	25,185 (2.3)	50 (2.2)	0.85
Coagulopathy	80,465 (7.3)	385 (16.6)	<0.001
Obesity	203,360 (18.5)	425 (18.4)	0.93
Weight loss	68,815 (6.3)	290 (12.5)	<0.001
Fluid and electrolyte disorders	450,515 (41)	1510 (65.2)	<0.001
Blood loss anemia	3,980 (0.4)	15 (0.6)	0.3
Deficiency anemia	42,750 (3.9)	145 (6.3)	0.008
Alcohol abuse	364,090 (33.1)	645 (27.9)	0.018
Drug abuse	93,740 (8.5)	235 (10.1)	0.22
Psychosis	13,545 (1.2)	40 (1.7)	0.33
Depression	157,780 (14.4)	325 (14)	0.84
Hypertension, complicated	119,175 (10.8)	945 (40.8)	<0.001

Outcomes 

**Figure 2:**
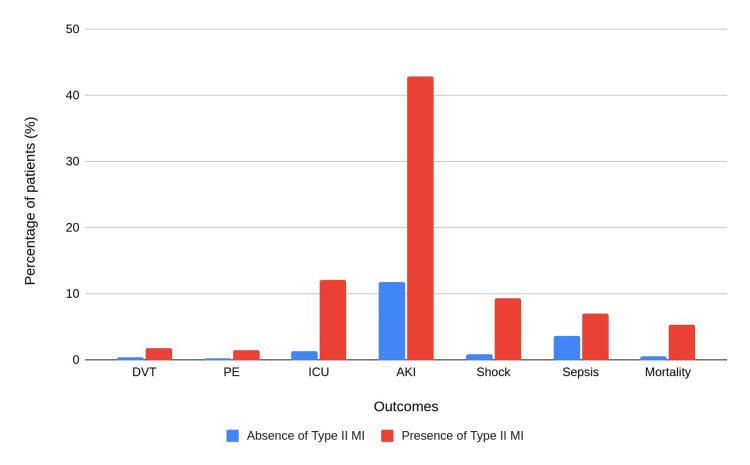
Outcomes in patients with and without type 2 MI MI: myocardial infarction; DVT: deep vein thrombosis: pulmonary embolism; ICU: intensive care unit; AKI: acute kidney injury All the outcomes were found to be statistically significant (p<0.001).

The outcomes, stratified by the presence of type 2 MI are presented in Table 3 and Figure 2.

**Table 3:** Outcomes of patients with type AP, stratified by type 2 MI MI: myocardial infarction; AKI: acute kidney injury; ICU: intensive care unit; PE: pulmonary embolism; DVT: deep vein thrombosis

Outcomes	Absence of Type 2 MI n (%)	Presence of Type 2 MI n (%)	p-value
Mortality	6,440 (0.6)	125 (5.4)	<0.001
Sepsis	40,665 (3.7)	165 (7.1)	<0.001
Shock	9,775 (0.9)	215 (9.3)	<0.001
AKI	129,660 (11.8)	995 (42.9)	<0.001
ICU	15,270 (1.4)	280 (12.1)	<0.001
PE	3,035 (0.3)	35 (1.5)	<0.001
DVT	4,615 (0.4)	45 (1.9)	<0.001

The results of the multivariate regression model to identify an association between type 2 MI and outcomes are presented in Table 4. 

**Table 4:** Results of multivariate regression to identify associations between type 2 MI and outcomes. MI: myocardial infarction; AKI: acute kidney injury; ICU: intensive care unit; PE: pulmonary embolism; DVT: deep vein thrombosis

Outcome	Odds Ratio	95% Confidence Interval	p-value
Death	2.4	1.5.3.8	<0.001
Sepsis	1.5	1.0-2.1	0.03
Shock	3.4	2.2-5.3	<0.001
AKI	2.5	2.0-3.1	<0.001
ICU	3.3	2.3-4.8	<0.001
PE	2.5	1.1-5.5	0.02
DVT	1.7	0.8-3.4	0.1
Length of stay	2.1	1.4-2.8	<0.001
Total hospitalization charges	45,088.10	30,224.1-59,952	<0.001

In-hospital Mortality

The presence of type 2 MI was associated with a significantly higher mortality rate of 5.4% compared to 0.6% in patients without type 2 MI. Multivariate analysis revealed that patients with type 2 MI had increased odds of death (adjusted odds ratio, aOR=2.4, 95% CI 1.5-3.8, p<0.001). 

Sepsis

The type 2 MI group had a higher rate of sepsis (7.1%) compared to the non-type 2 MI group (3.7%). Multivariate analysis demonstrated a statistically significant association between type 2 MI and sepsis development (aOR=1.5, 95% CI 1.0-2.1, p=0.03).

Shock

Shock was significantly more common in the type 2 MI group (9.3%) compared to patients without type 2 MI (0.9%). The multivariate analysis showed that patients with type 2 MI had a significantly higher risk of shock (aOR=3.4, 95% CI 2.2-5.3, p<0.001).

AKI

The prevalence of acute kidney injury (AKI) was significantly higher in the type 2 MI group (42.9%) compared to patients without type 2 MI (11.8%). Multivariate analysis revealed a significant association between type 2 MI and the development of AKI (aOR=2.5, 95% CI 2.0-3.1, p<0.001)

ICU Admission

A significantly higher proportion of type 2 MI patients (12.1%) required ICU admission compared to those without type 2 MI (1.4%). Multivariate analysis demonstrated that type 2 MI was independently associated with an increased risk of ICU admission (aOR=3.3, 95% CI 2.3-4.8, p<0.001).

PE

Pulmonary embolism (PE) was more prevalent in the type 2 MI group (1.5%) compared to patients without type 2 MI (0.3%). The multivariate analysis indicated a significant association between type 2 MI and the occurrence of PE (aOR=2.5, 95% CI 1.1-5.5, p=0.02).

DVT

Although not statistically significant, the type 2 MI group had a higher prevalence of deep vein thrombosis (DVT) (1.9%) compared to patients without type 2 MI (0.4%). The multivariate analysis did not show a significant association between type 2 MI and DVT (aOR=1.7, 95% CI 0.8-3.4, p=0.1).

Length of Stay (LOS)

The mean LOS in patients with type 2 MI was 8.02 days (+/-0.4), while the mean LOS in the other group was 4.2 (+/-0.1) days. The presence of type 2 MI was associated with a longer hospital stay. After adjusting for confounding factors, an independent association between type 2 MI and an increased length of stay (adjusted coefficient=2.1, 95% CI 1.4-2.8, p<0.001), indicating that patients with type 2 MI had a longer duration of hospitalization.

Total Hospitalization Charges

The mean hospitalization charges in patients with type 2 MI was $105,416 (+/-$7,903) while the mean hospitalization charges in the other group were 41,287.5 (+/-236.5). After adjusting for confounding factors, type 2 MI was independently associated with higher total hospitalization charges (adjusted coefficient-$45,088.1, 95% CI $30,224.1-$59,952, p<0.001), indicating that patients with type 2 MI incurred higher healthcare costs during their hospital stay.

## Discussion

Our study reported that only a small proportion of patients (0.002%) out of 1.1 million patients of AP were diagnosed with type 2 MI. Various mechanisms have been proposed to explain the rise in cardiac enzymes in AP. It is well known that pancreatic enzymes are responsible for tissue destruction and pseudocyst formation [21]. These enzymes may cross the diaphragm directly, or through the bloodstream or lymphatic flow, and lead to direct myocardial injury [21]. Inflammatory mediators released in AP could also lead to increased myocardial membrane permeability, catecholamine-induced microvascular damage, and coronary artery vasospasm [20,21]. Hypotension, electrolyte disturbances, and stress cardiomyopathy likely contribute as well [22]. Another explanation is that high enzyme levels might not necessarily indicate cardiac injury and may in fact be a result of rhabdomyolysis [23].

In our study, the presence of type 2 MI was associated with a 140% higher risk of in-hospital mortality. This is in line with existing literature [10]. Previous studies have observed a correlation between cardiac events and the severity of AP [6]. Various markers of cardiac injury have been linked to severe outcomes in patients with AP [9,15,21,24]. Raised levels of CK-MB have also been associated with higher mortality and development of severe AP, characterized by prolonged organ failure [15]. On the contrary, NT-proBNP levels have shown mixed results in predicting severity and organ failure among patients with AP [24]. Raised levels of troponin T have also been associated with poor prognosis [9]. While the mechanism of the increase in troponins in patients with pancreatitis is unknown, injudicious thrombolysis of pancreatitis patients with elevated troponin levels can produce disastrous outcomes [21].

Our study notes that patients with type 2 MI had higher odds of developing AKI. This can be attributed to pancreatitis being a hypovolemic state [22]. It has also been suggested in the past that the development of AKI in patients with pancreatitis can lead to worse outcomes and underscores the need for appropriate fluid resuscitation in these patients [25]. Early fluid resuscitation has been shown to prevent worse outcomes in pancreatitis. In critically ill septic patients, AKI is a frequent, devastating complication of severe AP and a significant predictor of morbidity and mortality [25]. Additionally, our study noted a higher incidence of sepsis in patients with type 2 MI. Sepsis could putatively precipitate cardiovascular events as reduced perfusion may precipitate demand ischemia [26]. Sepsis has been associated with both early and late mortality [26]. AKI is a common complication of sepsis and this might also be contributing to the higher incidence noted [27]. 

It has been suggested that thromboembolic events such as deep vein thrombosis (DVT) and pulmonary embolism (PE) can result in worse outcomes [27]. One study demonstrated that patients with pancreatitis who developed PE, and DVT had longer hospitalizations, more readmissions, and were at higher risk of mortality than those who did not [28]. The thrombotic events have been described in AP previously and can be explained by the widespread systemic inflammatory response [29]. PE is a rare but potentially fatal complication of AP and if not detected in time, the mortality rate is extremely high [29]. Our study noted a strong correlation between type 2 MI and PE. It is well known that PE can precipitate type 2 MI and thus any patient with elevated troponin and shortness of breath should be screened for PE and undergo appropriate imaging in order to make a timely diagnosis.

Our study notes that patients with type 2 MI had higher resource utilization as evidenced by higher odds of requiring ICU admission, longer length of stay, and higher resource utilization. This could be attributed to the higher severity of the disease in patients with type 2 MI as these patients had worse outcomes than their counterparts. It is also likely that these patients had extensive workup to rule out coronary artery disease, which might have affected resource utilization. Previous studies have concurred that patients with cardiovascular dysfunction have higher LOS when admitted for AP [30].

We recognize the limitations of our study. First, our study relies on ICD-10 codes and is prone to errors. Standard ICD-10 codes were used to reduce the errors. Second, longitudinal patient follow-up was not possible because data is collected only during hospitalization. The third drawback is the ambiguity inherent in the type 2 MI diagnosis itself. We excluded the patients with STEMI and NSTEMI to prevent their inclusion in any of the groups as it might have affected the analysis. The strengths of the study are its large sample size and the absence of selection bias, which is common in regional studies.

## Conclusions

Overall, we found that although the prevalence of type 2 MI is low in patients with AP, its development may be associated with higher morbidity and mortality. Patients with type 2 MI also faced a longer duration of hospital stay and higher costs of hospitalization. Future studies capturing more granular data are needed to investigate the cause as well as the outcomes among these patients. Physicians should be acutely aware of the possible catastrophic events in these patients and appropriate management with aggressive fluid resuscitation might be needed in a timely manner to prevent worse outcomes.
